# International Society of Sports Nutrition position stand: Nutrient timing

**DOI:** 10.1186/1550-2783-5-17

**Published:** 2008-10-03

**Authors:** Chad Kerksick, Travis Harvey, Jeff Stout, Bill Campbell, Colin Wilborn, Richard Kreider, Doug Kalman, Tim Ziegenfuss, Hector Lopez, Jamie Landis, John L Ivy, Jose Antonio

**Affiliations:** 1 Department of Health and Exercise Science, University of Oklahoma, Norman, OK 73019, USA; 2 Endocrinology and Diabetes Section, Department of Pediatrics, University of Oklahoma Health Sciences Center, Oklahoma City, OK 73104, USA; 3 Center for Physical Development Excellence, Department of Physical Education, United States Military Academy, 727 Brewerton Road, West Point, NY 10996, USA; 4 School of Physical Education & Exercise Science, University of South Florida, Tampa, FL 33620, USA; 5 Exercise & Sport Science Department, University of Mary-Hardin Baylor, Belton, TX 76513, USA; 6 Department of Health & Kinesiology, Texas A&M University, College Station, TX 77843, USA; 7 Nutrition/Endocrinology Division, Miami Research Associates, Miami, FL 33143, USA; 8 Division of Sports Nutrition and Exercise Science, The Center for Applied Health Sciences, Fairlawn, OH 44333, USA; 9 Department of Physical Medicine and Rehabilitation, Northwestern University Feinberg School of Medicine, Chicago, IL 60611, USA; 10 Department of Biology, Lakeland Community College, Kirtland, OH 44094, USA; 11 Department of Kinesiology & Health Education, University of Texas, Austin, TX 78712, USA; 12 Farquhar College of Arts and Sciences, Nova Southeastern University, Fort Lauderdale, FL 33314, USA

## Abstract

Position Statement: The position of the Society regarding nutrient timing and the intake of carbohydrates, proteins, and fats in reference to healthy, exercising individuals is summarized by the following eight points: 1.) Maximal endogenous glycogen stores are best promoted by following a high-glycemic, high-carbohydrate (CHO) diet (600 – 1000 grams CHO or ~8 – 10 g CHO/kg/d), and ingestion of free amino acids and protein (PRO) alone or in combination with CHO before resistance exercise can maximally stimulate protein synthesis. 2.) During exercise, CHO should be consumed at a rate of 30 – 60 grams of CHO/hour in a 6 – 8% CHO solution (8 – 16 fluid ounces) every 10 – 15 minutes. Adding PRO to create a CHO:PRO ratio of 3 – 4:1 may increase endurance performance and maximally promotes glycogen re-synthesis during acute and subsequent bouts of endurance exercise. 3.) Ingesting CHO alone or in combination with PRO during resistance exercise increases muscle glycogen, offsets muscle damage, and facilitates greater training adaptations after either acute or prolonged periods of supplementation with resistance training. 4.) Post-exercise (within 30 minutes) consumption of CHO at high dosages (8 – 10 g CHO/kg/day) have been shown to stimulate muscle glycogen re-synthesis, while adding PRO (0.2 g – 0.5 g PRO/kg/day) to CHO at a ratio of 3 – 4:1 (CHO: PRO) may further enhance glycogen re-synthesis. 5.) Post-exercise ingestion (immediately to 3 h post) of amino acids, primarily essential amino acids, has been shown to stimulate robust increases in muscle protein synthesis, while the addition of CHO may stimulate even greater levels of protein synthesis. Additionally, pre-exercise consumption of a CHO + PRO supplement may result in peak levels of protein synthesis. 6.) During consistent, prolonged resistance training, post-exercise consumption of varying doses of CHO + PRO supplements in varying dosages have been shown to stimulate improvements in strength and body composition when compared to control or placebo conditions. 7.) The addition of creatine (Cr) (0.1 g Cr/kg/day) to a CHO + PRO supplement may facilitate even greater adaptations to resistance training. 8.) Nutrient timing incorporates the use of methodical planning and eating of whole foods, nutrients extracted from food, and other sources. The timing of the energy intake and the ratio of certain ingested macronutrients are likely the attributes which allow for enhanced recovery and tissue repair following high-volume exercise, augmented muscle protein synthesis, and improved mood states when compared with unplanned or traditional strategies of nutrient intake.

## Nutrient timing and exercise: a review of the literature

### Introduction

Previous research has demonstrated that the timed ingestion of carbohydrate, protein, and fat may significantly affect the adaptive response to exercise. The overall concept of macronutrient ratio planning for the diets of athletes is not addressed directly within this position stand, as there is no one recommendation which would apply to all individuals. However, the ISSN refers the reader to the latest Institute of Medicine Guidelines for Macronutrient intake as a source of more general information [1]. The purpose of this collective position statement is to highlight, summarize, and assess the current scientific literature, and to make scientific recommendations surrounding the timed ingestion of carbohydrates (CHO), protein (PRO), and fat. The enclosed recommendations are suitable for researchers, practitioners, coaches and athletes who may use nutrient timing as a means to achieve optimum health and performance goals. This position stand is divided into three primary sections: pre-exercise, during exercise and post-exercise. Each section concludes with several bullet points that highlight the key findings from each of the areas.

## Nutrient timing: pre-exercise

Nutritional considerations prior to exercise have traditionally examined the administration of CHO to maximize endogenous glycogen stores [2-6] and maintain serum glucose levels during endurance exercise [4,7]. More recently, studies have begun to provide data supporting the contention that pre-exercise ingestion of CHO, amino acids, PRO, and creatine (Cr) prior to resistance training are effective modalities for enhancing exercise training adaptations [8-12] and decreasing exercise associated muscle damage [12,13].

### Pre-exercise ingestion of carbohydrate

Body stores of glycogen are limited [7,14], and will last a few hours at best during moderate to high intensity levels (65 – 85% VO_2_max) of exercise [15]. As glycogen levels diminish, exercise intensity, and work output decrease [14], and frequently muscle tissue breakdown and immunosuppression ensues [16,17]. Due to the well-established connection between negative body changes and the depletion of glycogen stores, the concept of CHO loading is likely the oldest form of all the nutrient timing practices. Daily ingestion of high-CHO meals (~65% CHO) is recommended to maintain muscle glycogen, while increased ingestion rates are employed (~70% CHO) in the 5 – 7 days leading up to competition as a means of maximizing muscle and liver glycogen stores and in order to sustain blood glucose during exercise [2,4,5]. Traditional CHO loading studies utilized a glycogen depletion phase typically lasting 3 – 6 days prior to increasing CHO intake [2-5,18]. Maximal levels of glycogen storage, however, may be achieved after just 1 – 3 days of consuming a high-CHO diet while minimizing physical activity [2,4]. For example, Kavouras and colleagues instructed twelve endurance-trained cyclists to perform a 45 min bicycle ride at 82% VO_2 _peak after six days of following either an isoenergetic high-CHO (600 g) or low-CHO (100 g) diet. Prior to exercise, muscle glycogen levels were significantly higher (p < 0.05) in the high-CHO condition (104.5 ± 9.4 mmol/kg/wet wt) when compared to the low-CHO condition (72.2 ± 5.6 mmol/kg/wet wt). Serum glucose levels increased during exercise in the high-CHO condition with no changes evident in the low-CHO condition. Finally, post-exercise glucose levels were also significantly greater for the high-CHO condition when compared to the low-CHO condition, suggesting that individuals subjected to the high-CHO condition were better able to sustain blood glucose levels. No changes were noted for serum free fatty acids, triglycerides or insulin (p > 0.05) [4]. Another study by Bussau et al. [2] found that eating a high-glycemic CHO (10 g/kg/day) diet for as little as one day could significantly increase muscle glycogen levels. In that particular study, muscle glycogen levels increased from baseline levels of 95 ± 5 mmol/kg/wet wt to 180 ± 15 mmol/kg/wet wt after one day, and remained at those levels for three subsequent days.

Research involving the ingestion of single high CHO feedings has also demonstrated the promotion of higher levels of muscle glycogen and an improvement of blood glucose maintenance (euglycemia), though changes in performance have been equivocal [14,19-22]. In a study completed by Coyle et al. [14], cyclists were instructed to ingest a high CHO meal four hours prior to completing a prolonged (105 min) exercise bout at 70% VO_2_max. The single meal increased muscle glycogen by 42%, which resulted in higher levels of CHO oxidation and utilization of muscle glycogen. In contrast, Febbraio et al. [21] reported that ingestion of a high-glycemic meal 45 min before 135 min of cycling exercise was not responsible for changes in muscle glycogen utilization or performance when compared to a low-glycemic meal or water. A follow-up study in 2000 found no changes in performance after 150 min of cycling at 70% VO_2_max when either a high-glycemic or low-glycemic meal was consumed 30 min before exercise [20]. Earnest et al. compared the effects of the pre-exercise ingestion of honey (low-glycemic), dextrose (high-glycemic) and a placebo over a 64-kilometer time trial in a crossover fashion. While CHO ingestion was thought to be responsible for greater power output over the last 16% of the time trials, no difference in performance was noted between the high and low-glycemic groups [19]. In general, research involving CHO ingestion within an hour prior to exercise demonstrates equivocal results regarding changes in performance, but studies have routinely shown the ability of CHO ingestion to maximize glycogen utilization and promote CHO oxidation. Hawley and Burke [22] summarized several studies that administered some form of CHO within one hour prior to exercise: one study reported a decrease in performance [23], three studies reported an increase in performance [24-26] and five studies reported no effect [21,27-30] (Additional File 1).

### Pre-exercise ingestion of amino acids and protein

Researchers have begun to explore the potential of ingesting PRO and/or amino acids, either alone or in combination with CHO, in order to enhance training adaptations to resistance exercise. An study investigating this potential relationship compared the ingestion of a CHO + PRO supplement (35 grams of CHO with 6 grams of essential amino acids) consumed either immediately before, or immediately after, a single bout of resistance exercise at 80% one-repetition maximum (1 RM) [9]. The authors concluded that the effect on the net PRO status (breakdown vs. synthesis) was greater when the supplement was ingested before exercise. They speculated that the increased serum amino acid levels present when tissue blood flow levels were significantly increased, likely led to an increase in PRO synthesis [9]. The same authors subsequently compared the changes in PRO metabolism following the ingestion of 20 grams of whey PRO both immediately before, or immediately after, a single resistance exercise bout at 80% 1 RM [31]. In this case the authors concluded that a pro-anabolic response was found when the whey PRO was ingested both before and after resistance exercise, but no differences were found between the two administration times [31]. Findings from these studies suggest that ingestion of amino acids and CHO, or whey PRO, before resistance exercise can maximally stimulate PRO synthesis after completion of the exercise bout [9,31].

Many studies have explored the use of pre-exercise PRO and CHO ingestion in preventing acute exercise-induced muscle damage [13], as well as the damage that may occur during prolonged periods of regular resistance training [8,10-12,32]. A recently published study evaluated 27 adult male participants who consumed either a placebo (a non-caloric sweetener), or a CHO + PRO solution (75 g CHO + 23 g PRO) 15 min before, or 15 min after completing a potentially muscle-damaging bout of eccentric contractions. Although the authors reported that the level of the muscle damage marker creatine kinase had increased and maximal force production of the muscle was reduced, the administration or timing of the nutrients did not appear to alter these markers of muscle damage [13]. In another study, participants ingested either a multi-nutrient (CHO + PRO + Fat) supplement or an isoenergetic maltodextrin placebo for seven days before reporting to the laboratory for two consecutive days of resistance training [12]. On both exercise days, the supplement was ingested 30 min prior to beginning the exercise bout. The multi-nutrient supplementation significantly improved vertical jump power and number of repetitions performed at 80% 1 RM. Additionally, multi-nutrient supplementation significantly increased serum levels of both growth hormone and free and total testosterone during and after the exercise bouts [12]. These latter findings suggest that pre-exercise ingestion may also create a favorable anabolic hormone environment. In another study involving unilateral resistance training, pre-exercise supplementation of whey PRO and leucine resulted in greater increases in maximal strength [11]. Thirty-three male participants completed six weeks of unilateral lower body resistance training while assigned to either a resistance training only (control) group, a resistance training + 26 g CHO (placebo) group, or a resistance training + 20 g whey PRO + 6 g leucine group. The authors concluded that pre-exercise supplementation of whey PRO + leucine promoted significantly greater increases in strength (+ 30.3%) when compared to the energy-matched placebo (+ 22.4%) and control (+ 3.6%) groups [11]. Two additional studies also compared the ingestion of CHO + PRO before and after eight and 12 weeks of resistance training, respectively. One study compared the pre-exercise and post-exercise ingestion of 1.2 g/kg whey PRO + 0.3 g/kg CHO, 1.2 g/kg soy PRO + 0.3 g/kg CHO, or placebo during eight weeks of resistance training. The authors found that PRO supplementation significantly increased strength and lean mass when compared to placebo, but no differences were found between the two forms of PRO [32]. The second study had participants perform heavy resistance exercise (3 sets of 6 – 8 repetitions at 85 – 90% 1 RM) 4 days per week for 10 weeks [10]. Participants were assigned to ingest either 20 g PRO (14 g whey and casein PRO + 6 g free amino acids), or 20 g CHO before and after each exercise bout for a total of 40 g/d of PRO or 40 g/d CHO. Individuals consuming the protein supplement experienced greater increases in body mass, fat-free mass, strength, serum levels of IGF-1, and intramuscular levels of IGF-1 mRNA, myosin heavy chain I and IIa expression, and myofibrillar protein content [10]. Collectively, the last two studies mentioned provide additional support for the concept that ingesting PRO before and after exercise can promote a greater training adaptation than consuming only an isoenergetic CHO placebo [10,32].

A 2006 study by Cribb and Hayes [8] used two different feeding strategies to determine the impact of nutrient timing, in regards to an exercise bout, for changes in strength, muscle hypertrophy and body composition. Participants were instructed to ingest equal quantities of a supplement containing PRO, Cr and CHO at a dose of 1 g/kg either immediately before and immediately after each workout, or in the morning and evening of each workout day. Significantly greater increases in lean body mass, 1 RM strength, type II muscle fiber cross-sectional area, and higher muscle Cr and glycogen levels were found when the supplements were consumed immediately before and after workouts [8]. In summary, ingestion of amino acids or PRO, either alone or in combination with CHO, in close temporal proximity to a bout of resistance exercise, appears to significantly increase muscle PRO synthesis [9,31]. Furthermore, adopting this strategy during a resistance training program results in greater increases in 1 RM strength and a leaner body composition [8,10-12,32].

### Summary of pre-exercise nutrient ingestion findings

• Glycogen stores are limited and depend largely on the nutritional status and the intensity and training level of the athlete [7,14]. Endogenous glycogen stores during moderate to high intensity levels (65 – 85% VO_2_max) of exercise may only last from 90 min to 3 h [15].

• Exercise intensity, pace and work output decrease as glycogen levels diminish [14]. Depletion of glycogen is associated with increased levels of muscle tissue breakdown and suppression of the immune system [16,17].

• Maximal endogenous glycogen stores are best promoted by following a high-glycemic, high-CHO diet (600 – 1000 grams or ~8 – 10 g/kg/d) [2,3,15].

• The optimal CHO and PRO content of a pre-exercise meal is dependent upon a number of factors including exercise duration and fitness level, but general guidelines recommend ingestion of 1 – 2 grams CHO/kg and 0.15 – 0.25 grams PRO/kg 3 – 4 hours before competition [15].

• Pre-exercise ingestion of essential amino acids or PRO alone increases muscle protein synthesis. In addition, ingesting PRO + CHO pre-exercise has been shown to produce significantly greater levels of muscle protein synthesis [9,31].

• Regular ingestion of various PRO sources in conjunction with CHO stimulates greater increases in strength and favorably impacts body composition when compared to CHO alone [8,10,11].

## Nutrient timing: during exercise

Much like the consideration of pre-exercise nutrient supplementation, a majority of the literature which has examined the impact of nutrient administration during exercise has focused on aerobic exercise [33-36], with a lesser emphasis on nutrient administration during resistance exercise [37-41].

### Glucose administration during endurance exercise

The initial research which dealt with nutrient administration during exercise scrutinized the optimal delivery of CHO in an effort to sustain blood glucose. For example, Australian researchers had eight highly-trained cyclists complete two trials at 70% VO_2_max until the point of volitional fatigue [42]. Before exercise, and every 15 min throughout, participants were either given a placebo or an 8% CHO solution to ingest. Ingestion of the CHO solution was associated with a 30% increase in time to reach volitional exhaustion, or a 47 min longer period of cycling when compared to placebo [42]. Widrick and colleagues [35] had participants complete 70 km of self-paced time trials under four different conditions: 1.) high glycogen (180.2 ± 9.7 mmol/kg/wet wt) + CHO beverage; 2.) high glycogen (170.2 ± 10.4 mmol/kg/wet wt) + Non-CHO beverage; 3.) low glycogen (99.8 ± 6.0 mmol/kg/wet wt) + CHO beverage; 4.) low glycogen (109.7 ± 5.3 mmol/kg/wet wt) + non-CHO beverage [35]. The CHO drink was ingested at the onset of exercise and every 10 km after, providing 116 ± 6 g CHO/trial. CHO administration maintained blood glucose, while blood glucose declined significantly under the non-CHO conditions. Over the final 14% of the time trial (9.8 km), power output and pace were significantly less in the low glycogen + non-CHO condition when compared to the other three conditions. Results from this study suggest exogenous CHO delivery during training is not as important if baseline glycogen levels are high, and if glycogen levels are low, CHO ingestion during endurance exercise will likely improve performance. In a similar investigation, nine trained athletes consumed both a CHO and a non-CHO control solution while completing a 90 min bout of high-intensity intermittent running [34]. The CHO solution was 6.9% CHO and was first provided immediately prior to exercise, and subsequently every 15 min after the exercise bout started. When CHO was ingested the participants were able to run significantly longer when compared to the control condition, providing additional evidence that CHO availability may be important for continued exercise performance [34]. An additional study highlighting the importance of CHO delivery during endurance exercise was completed by Febrraio et al. in 2000 [33]. This study, like several in this investigative field, utilized trained cyclists as participants. The cyclists undertook a 120 min bout of cycling at 63% of their peak power under four conditions: 1) placebo before and during exercise [PP]; 2) placebo 30 min before + CHO (2 g/kg in a 6.4% CHO solution) during exercise [PC]; 3) CHO (2 g/kg in a 25.7% CHO solution) before exercise + placebo during exercise [CP]; or 4) CHO (2 g/kg in a 25.7% CHO solution) before exercise + CHO (2 g/kg in a 6.4% CHO solution) [CC] during exercise. Blood glucose appearance and disappearance, and time trial performance was greater in the CC and PC trials when compared to the PP condition. The authors concluded that pre-exercise ingestion of CHO improves performance only when CHO ingestion is maintained throughout exercise, and ingestion of CHO during 120 min of cycling improves subsequent time trial performance [33]. Similarly, a study by Fielding et al. reported that more frequent intake of CHO (10.75 g CHO in 200 ml water; ~5% CHO solution) at 30 min intervals versus large feedings (86 gram doses) at 60 min intervals over a four hour bike ride equally sustained blood glucose and insulin activity, but the shorter interval of intake facilitated a significantly longer sprint ride to exhaustion at the end of exercise [43]. These findings conflicted with those of Burke et al. [44] who reported no impact of a glycemic meal consumed prior to exercise on subsequent time trial performance. Lastly, a 2007 study investigated the ability of a consumed CHO-gel preparation to maintain blood glucose levels and enhance performance during a high-intensity intermittent run in soccer players [45]. As with previous studies that have used CHO solutions, the CHO-gel promoted higher levels of blood glucose and facilitated improved performance in the intermittent bout of running when compared to the placebo [45]. In summary, the weight of evidence suggests that the ingestion of CHO during endurance type exercise is a well-established strategy to sustain blood glucose levels, spare glycogen [6], and potentially promote greater levels of performance. The interested reader is encouraged to consult the following reviews [15,46-49].

### Mixing carbohydrates to increase carbohydrate oxidation

A fairly novel area of research has examined the impact of mixing various forms of CHO in an effort to promote greater levels of CHO oxidation during prolonged exercise. It is well accepted that peak rates of CHO oxidation are commonly around 1 gram of CHO per minute or 60 grams per hour [15,48]. An increase in exogenous CHO availability, and subsequent oxidation, will result in improved maintenance of blood glucose and less reliance on liver and muscle glycogen stores. For example, recent studies have illustrated a 21% increase in CHO oxidation to 1.2 g CHO/min after ingesting a mixture of glucose and sucrose [50]; while a combination of maltodextrin and fructose was responsible for a 40% increase in peak CHO oxidation levels to approximately 1.5 g/min over maltodextrin alone during prolonged cycling at 60 – 65% VO_2_max [51]. Indeed, findings from this research team have regularly reported enhanced CHO oxidation rates, from 1.2 – 1.75 grams of CHO per minute [50,52-55]. Most recently, a 2008 paper by this same research group reported an 8% increase in time-trial performance after a 120 min ride at 55% maximum watts when ingesting a combination of glucose and fructose during exercise [56]. It should be noted that fructose is not as often used as a CHO supplement due to the potential for gastrointestinal upset.

### Adding protein or amino acids to carbohydrate during endurance exercise

The addition of PRO to CHO during exercise has also been investigated as a means to improve performance and facilitate recovery. In one study, participants completed 3 h of cycling @ 45 – 75% VO_2_max, followed by a time to exhaustion trial at 85% VO_2_max. During each session, participants consumed either a placebo, a 7.75% CHO solution, or a 7.75% CHO/1.94% PRO solution. While the CHO only group increased time to exhaustion (19.7 ± 4.6 min) versus the placebo (12.7 ± 3.1 min), the addition of PRO resulted in even greater performance (26.9 ± 4.5 min) [57]. A study by Saunders et al. examined the impact of a CHO + PRO combination for its ability to improve performance and minimize muscle damage [58]. Cyclists exercised to exhaustion on two different occasions separated by 12 – 15 h. During exercise, all participants ingested a 7.3% CHO solution, or a 7.3% CHO/1.8% PRO solution, every 15 min during exercise, and after exercise. CHO intake levels were the same for each group, although the total caloric intake was different (due to the energy supplied by the added PRO). A 29% increase in performance occurred after the first bout of exercise, and a 40% increase in performance after the 2^nd ^bout of exercise for the CHO + PRO group when compared to the CHO group. Additionally, post-exercise levels of muscle damage markers were 83% lower, suggesting the CHO + PRO supplement helped to attenuate the muscle damage associated with prolonged and exhaustive exercise [58]. A 2007 study by the same research group used a similar study design with a CHO + PRO gel during exercise and found that the gel again improved performance by 13% over a placebo [59]. Furthermore, a 2004 study recruited ultra-endurance athletes to compare the impact of CHO and CHO + PRO on changes in protein turnover and recovery after 6 h of endurance exercise [36]. PRO balance was negative during the CHO condition, but these findings were partially reversed (protein balance was still negative, but to a lesser degree) when PRO was added to the supplement. The authors concluded that combined ingestion of PRO and CHO improves net PRO balance at rest, as well as during exercise and post-exercise recovery [36].

### Addition of protein, amino acids and carbohydrate during resistance exercise

Delivering nutrients during single bouts of resistance exercise has been used to determine their impact on changes in muscle glycogen [40], mitigation of muscle damage [13,37], and promotion of an anabolic response [38,39,41]. Over the course of an estimated 40 min resistance training workout using the lower body, 1.0 g CHO/kg was provided before exercise and 0.5 g CHO/kg was given every 10 min throughout the workout to determine changes in muscle glycogen [40]. Decrements in muscle glycogen were offset by 49% when CHO was provided before and during the resistance exercise. The authors concluded that CHO supplementation before and during resistance exercise can maintain muscle glycogen stores and enhance the benefits of training [40].

Nutrient feedings during exercise have also been researched for their ability to offset muscle damage after intense resistance training [37]. Baty and colleagues[37] had 34 males complete an acute bout of heavy resistance training (3 sets × 8 reps @ 90% 1 RM) while consuming either a CHO solution (6.2% CHO) or a CHO + PRO solution (6.2% CHO + 1.5% PRO) before, during, and after the exercise bout. While no changes in performance were noted, the authors did report significantly greater levels of the anabolic hormone insulin and significantly lower levels of the catabolic hormone cortisol in the participants who ingested the CHO + PRO solution when compared to the CHO solution at several points after exercise. Furthermore, serum levels of myoglobin were lower during and immediately following exercise and creatine kinase was significantly lower 24 hours post exercise when the CHO + PRO supplement was provided. The authors concluded that the CHO + PRO solution had no impact over performance, but did lower serum markers of muscle damage during and several hours after completion of resistance training [37].

Two studies explored the changes in protein degradation and increases in serum levels of cortisol with ingestion of a CHO + PRO solution (6% CHO + 6% essential amino acids) during a single bout of resistance training exercise [38,39]. During both studies, 32 participants completed a 60 min bout of resistance training while consuming either a 6% CHO solution, a 6% CHO + 6 g essential amino acid (EAA) solution, or a placebo beverage. Serum levels of cortisol increased 105% in the placebo group, while changes in the CHO and CHO + EAA groups were 11% and 7%, respectively. Further, urinary levels of 3-methyl-histidine (a marker of muscle protein breakdown) were reduced by 27% in the CHO + EAA group, while these values increased by 56% in the placebo group [38,39]. The authors concluded that the suppression of PRO breakdown and cortisol levels may help to promote accretion of muscle PRO with prolonged periods of resistance training and supplementation. Their final study examined the influence of a 12 week resistance training program in combination with CHO and EAA supplementation. In conjunction with the two previous studies, a 6% CHO solution, 6% CHO + 6 g EAA solution, or a placebo was consumed during resistance exercise. Serum insulin and cortisol, urinary markers of PRO breakdown, and muscle cross-sectional area were measured [41]. CHO + PRO ingestion corresponded with a 26% decrease in markers of PRO breakdown, while the placebo group increased PRO breakdown by 52%. Furthermore, muscle cross-sectional area of the type I, IIa and IIb fibers were increased with the CHO + PRO group, which displayed the greatest gains relative to placebo. The authors concluded that CHO + PRO supplementation with prolonged resistance training enhances muscle anabolism when compared to either CHO alone, or a placebo, by maximizing the anabolic response and attenuating the catabolic response [41]. Similarly, a 2008 study by Beelen et al. [60] had participants ingest a bolus of CHO + PRO at a dose of 0.15 g/kg body wt before initiating and at 15 min intervals during a two-hour bout of resistance training. PRO + CHO lowered the rate of PRO breakdown by 8.4 ± 3.6% and increased fractional PRO synthesis by 49 ± 22%, resulting in a 5-fold increase in PRO balance. Overall, the research supports the conclusion that the intake of nutrients such as CHO alone, or a combination of CHO + PRO, during resistance training may help promote greater levels of muscle glycogen, increase muscle cross-sectional area, and decrease PRO breakdown [38-41].

### Summary of during exercise nutrient findings

• CHO availability during exercise and muscle glycogen levels are major determinants of endurance performance. CHO administration becomes even more important when muscle glycogen levels are low at the onset of exercise [35,42].

• As exercise duration increases beyond 60 min, exogenous sources of CHO become important to maintain blood glucose and muscle glycogen stores. This CHO source should supply 30 – 60 grams of CHO per hour and can typically be delivered by drinking 1 – 2 cups of a 6 – 8% CHO solution (8 – 16 fluid ounces) every 10 – 15 minutes [49].

• Mixing different forms of CHO has been shown to increase muscle CHO oxidation from 1.0 g CHO/min to levels ranging from 1.2 g – 1.75 g CHO/min [50,52-54]; an effect which is associated with an improvement in time trial performance [56].

• Glucose, fructose, sucrose and maltodextrin can be used in combination, but large amounts of fructose are not recommended due to the greater likelihood of gastrointestinal problems.

• The addition of PRO to CHO at a ratio of 3 – 4:1 (CHO: PRO) has been shown to increase endurance performance during both acute exercise and subsequent bouts of endurance exercise [57,58].

• Ingesting CHO alone, or in combination with PRO, during resistance exercise increases muscle glycogen stores [40], offsets muscle damage [37], and facilitates greater training adaptations after acute [38,39] and prolonged periods of resistance training [41].

## Nutrient timing: post-exercise

Many nutritional interventions have been considered to enhance recovery from exercise. The body of published research supports the practice of ingesting nutrients to enhance performance for both endurance and resistance training athletes. There is also sound evidence which supports the value of post-exercise nutritional supplementation as a means of improving the recovery of intramuscular glycogen, providing a positive stimulation for acute changes in amino acid kinetics and improvement of the net PRO balance, as well as enhancing the overall adaptation to resistance training.

### Maximization of muscle glycogen re-synthesis

Athletes who ingest 1.5 g CHO/kg body wt. within 30 minutes after exercise have been shown to experience a greater rate of muscle glycogen re-synthesis than when supplementation is delayed by two hours, largely due to a greater sensitivity of muscle to insulin [61]. Additionally, both solid and liquid forms of CHO promote similar levels of glycogen re-synthesis [15,62,63]. Moreover, different forms of CHO have different effects on insulin levels, with fructose ingestion being associated with lower levels of glycogen re-synthesis than other forms of simple carbohydrates [64]. It has been demonstrated that delaying CHO ingestion by as little as two hours can reduce the rate of muscle glycogen re-synthesis by 50% [61]. If an athlete is glycogen-depleted after exercise, a CHO intake of 0.6 – 1.0 g CHO/kg/h during the first 30 minutes, and again every two hours for 4 – 6 hours, can adequately replace glycogen stores [65,66]. Similarly, maximal glycogen re-synthesis rates have been achieved when 1.2 g CHO/kg/h is consumed every 15 – 30 minutes [65,67]. Consequently, frequent feedings of CHO in high amounts over the 4 – 6 hours following exercise is recommended to ensure recovery of muscle and liver glycogen [15,49]. Additional studies have also reported that maximal glycogen levels can be restored within 24 h if optimal levels of CHO are available (8 g CHO/kg/day), and the degree of glycogen depletion is not too severe [62]. A CHO intake of 9 – 10 g CHO/kg/day is suggested for athletes who are completing intense exercise bouts on consecutive days [68].

Several studies have suggested that adding PRO to CHO supplementation after exercise may help to promote greater recovery of muscle glycogen and attenuate muscle damage. Ivy and colleagues [69] instructed cyclists to complete a 2.5 h bout of intense cycling before ingesting either a CHO + PRO + Fat (80 g CHO, 28 g PRO, 6 g Fat), low CHO (80 g CHO, 6 g fat), or a high CHO (108 g CHO, 6 g fat) supplement immediately after exercise, and 2 h post-exercise, to determine if the CHO + PRO + Fat combination promoted greater restoration of muscle glycogen. While glycogen replenishment did not differ between the two CHO conditions (low CHO [70.0 ± 4.0 mmol/kg/wet wt] and high CHO [75.5 ± 2.8 mmol/kg/wet wt]), muscle glycogen levels were significantly greater (p < 0.05) in the CHO + PRO + Fat treatment (88.8 ± 4.4 mmol/kg/wet wt). The authors concluded that a CHO + PRO + Fat supplement may be more effective because of its provocation of a more pronounced insulin response [66,69,70]. Similarly, studies by Berardi and Tarnopolsky [71,72] utilized cyclists for the completion of exercise bouts of 60 – 90 min on separate occasions before ingesting CHO + PRO or CHO alone. Both authors concluded that ingestion of either CHO preparation resulted in greater restoration of muscle glycogen when compared to a placebo. Berardi [71], however, reported even greater glycogen levels when the CHO + PRO combination was consumed post-exercise. Furthermore, the availability of essential amino acids (EAA) following exercise, especially the branched-chain amino acids, have been reported to influence recovery by optimizing PRO re-synthesis as well as glycogen re-synthesis rates after exercise [61,69,70,72-74]. As these studies suggest, the ingestion of CHO (1 – 1.5 g CHO/kg/day) within 30 minutes following the termination of an exercise bout promotes restoration of muscle glycogen, while the addition of PRO may have additional benefits in enhancing both muscle PRO and glycogen re-synthesis.

### Acute changes in amino acid kinetics and protein balance

A single bout of resistance training modestly stimulates PRO synthesis, but also further stimulates PRO breakdown resulting in an overall negative PRO balance after exercise [75,76]; an effect which shifts PRO balance more towards neutral as training status progresses [76]. Infusion or ingestion of amino acids increases amino acid concentrations at rest or after resistance exercise [77]. In addition, providing CHO in combination with amino acids immediately before or after exercise may further increase amino acid availability and post-exercise PRO synthesis [73,78]. Consequently, increasing the concentration and availability of amino acids in the blood is an important consideration when attempting to promote increases in lean tissue and improve body composition with resistance training [77,79].

Ingestion of a large dose of CHO (100 g) alone and within 1 h after resistance exercise causes marginal improvements in overall PRO synthesis while maintaining a negative net PRO balance [78]. While no studies have found CHO to be detrimental, it is not the ideal nutrient (in isolation) to consume after resistance exercise. Its inclusion, however, is an important consideration regarding stimulation of glycogen re-synthesis and enhanced palatability [69,72]. The EAAs, however, in dosages ranging from 6 – 40 grams have routinely been shown to play a primary role in promoting muscle PRO synthesis [74,80], though adding CHO to them may enhance this effect [9,81].

Regarding post-exercise timing, ingestion of amino acids after resistance exercise has been shown at many different time points to stimulate increases in muscle PRO synthesis, cause minimal changes in PRO breakdown and increase overall PRO balance [74,75,80]. Unfortunately, the optimal time point for supplementation has not yet been demonstrated. Similar changes have been found in studies that have administered amino acids alone, or with CHO, immediately, 1 h, 2 h and 3 h after exercise [9,74,79,81]. Levenhagen et al. [82] found that after ingesting 10 g PRO + 8 g CHO + 3 g Fat either immediately or 3 h after 60 min of moderate-intensity exercise, leg muscle glucose uptake and whole body glucose utilization were elevated threefold and 44%, respectively. Leg muscle and whole-body PRO synthesis was increased threefold and 12%, respectively. Furthermore, Tipton and colleagues [9] supplemented participants with 35 g sucrose + 6 g EAAs immediately before, and immediately after, a single bout of resistance exercise. They reported significantly greater levels of PRO synthesis when the nutrients were ingested immediately before the exercise bout.

In summary, the optimal dosage and ratio of EAAs and CHO necessary to optimize protein balance is not currently known. Studies using similar techniques to measure protein kinetics during resistance exercise have used 6 g EAA only, 6 g EAA + 6 g non-essential amino acids, 12 g EAA only, 17.5 g whey PRO, 20 g casein PRO, 20 g whey PRO, 40 g mixed amino acid, and 40 g EAA only; all have noted similar increases in PRO synthesis and PRO balance [9,73,77]. While the ratio of CHO to PRO requires additional investigation, a often utilized practical approach is to consume a supplement containing CHO + PRO in a 3:1 or 4:1 ratio within 30 minutes following exercise, which translates to 1.2 – 1.5 g/kg of simple CHO (e.g., dextrose, sucrose) with 0.3 – 0.5 g/kg of a quality PRO containing EAA [73,74,83]. A summary of relevant findings is provided in Table 2 (Additional File 2).

### Post-exercise supplementation for promotion of training adaptations

In an attempt to stimulate greater adaptations associated with resistance training researchers have investigated the impact of administering varying combinations of CHO and PRO after (1 – 3 h post-exercise) each exercise bout over the course of training [8,10,32,84-91]. The collective findings of these studies support the rationale for post-exercise administration of CHO and PRO to facilitate greater improvements in strength and body composition. Additionally, PRO source may be an important consideration as studies have suggested that whey PRO may exhibit a faster kinetic digestive pattern when compared to casein PRO [92,93]. Furthermore, this faster kinetic pattern for whey PRO is responsible for greater increases in PRO synthesis upon ingestion, with little to no impact over PRO breakdown. Casein PRO, on the other hand, releases its amino acids at a slower rate from the gut. This kinetic pattern results in little control over PRO synthesis, but a powerful attenuation of PRO breakdown. When both of these milk PRO sources are compared using area under the curve analysis, results suggest that casein may be responsible for a greater overall improvement in PRO balance when compared to whey [92,93]. A summary of these studies is provided below in table 3 (Additional File 3), but the universal findings of these studies suggest that adding some combination of CHO (50 – 75 g) to a PRO source (20 – 75 g) while completing heavy resistance training facilitates an increase in the development of lean mass and overall improvements in body fat %.

### Adding creatine to carbohydrate and protein

In addition to providing a combination of CHO + PRO after regular resistance training, researchers have also examined the impact of adding creatine monohydrate (Cr) in an attempt to facilitate greater training adaptations [84,85,88,90]. Cr is a popular dietary supplement that has been heavily researched for its ability to increase performance and facilitate positive training adaptations [94,95]. For example, Tarnopolsky et al. [90] had previously untrained male participants undergo resistance training for eight weeks while ingesting, in a double-blind fashion, either a Cr (10 g) + CHO (75 g) or PRO (10 g) + CHO (75 g) combination 30 min after exercise. Following assessment of changes in strength and muscle mass, the Cr + CHO group gained significantly more body mass (5.4% increase from baseline) when compared to the PRO + CHO group (2.4% increase). Changes in fat-free mass, muscle fiber area, 1 RM, and isokinetic strength improved in both groups, but were not different among groups. Another study had participants resistance train for 11 weeks while consuming daily one of the following: 1) 0.1 g Cr/kg/day + 1.5 g CHO/kg/day, 2) 0.1 g Cr/kg/day + 1.5 g whey PRO/kg/day), 3) 1.5 g/kg/d whey PRO only or 4) 1.5 g CHO/kg/day only. Supplementation in the first three groups resulted in greater increases in 1 RM strength and muscle hypertrophy when compared to CHO only, but no differences were found among the groups ingesting Cr in conjunction with either CHO or PRO [85].

In contrast, two published studies have suggested that the addition of Cr may be responsible for greater increases in muscle hypertrophy. The first study had participants complete heavy resistance training for 10 weeks while ingesting one of the following isoenergetic groups: 1) 1.5 g/kg/day of PRO only, 2) 0.75 g PRO/kg/day + 0.75 g CHO/kg/day, or 3) 0.1 g Cr/kg/day + 0.75 g PRO/kg/day + 0.75 g CHO/kg/day. Changes in strength and muscle hypertrophy were found to be greater in the Cr + CHO + PRO group when compared to the CHO + PRO group [84]. Similarly, Kerksick and colleagues [88] had participants complete 12 weeks of resistance training while ingesting a blend of whey and casein PRO, with or without Cr. While all groups saw increases in strength and muscle mass, those groups ingesting Cr with the PRO blend experienced greater gains in body mass and fat-free mass. Though these findings are somewhat mixed, the available data does provide support that adding Cr to a post-exercise regimen of CHO and PRO may help to facilitate greater improvements in body composition during resistance training [84,85,88,90].

### Summary of post-exercise nutrient ingestion findings

• Post-exercise (within 30 minutes) consumption of CHO at high dosages (8 – 10 g CHO/kg/day) has been shown to stimulate muscle glycogen re-synthesis [15,65].

• Adding PRO (0.2 g – 0.5 g PRO/kg/day) to CHO at a ratio of approximately 3: 1 (CHO: PRO) has been shown to stimulate glycogen re-synthesis to a greater extent [69].

• Post-exercise ingestion (immediately after through 3 hours post) of amino acids, primarily EAAs, have been shown to stimulate robust increases in muscle PRO synthesis [73,74,83]. The addition of CHO may increase PRO synthesis even more, while pre-exercise consumption may result in the best response of all [9].

• During prolonged resistance training, post-exercise consumption of CHO + PRO supplements in varying amounts have been shown to stimulate improvements in strength and body composition when compared to control, placebo, or CHO-only conditions [10,87,90].

• The addition of Cr (0.1 g Cr/kg/day) to a CHO + PRO supplement may facilitate even greater adaptations to resistance training [84,88].

## Conclusion

The scientific literature associated with nutrient timing is an extremely popular, and thus ever-changing, area of research. Upon reviewing the available literature, the following conclusions can be drawn at this point in time:

• Prolonged exercise (> 60 – 90 min) of moderate to high intensity exercise will deplete the internal stores of energy, and prudent timing of nutrient delivery can help offset these changes.

• During intense exercise, regular consumption (10 – 15 fl oz.) of CHO/electrolyte solution delivering 6 – 8% CHO (6 – 8 g CHO/100 ml fluid) should be consumed every 15 – 20 min to sustain blood glucose levels.

• Glucose, fructose, sucrose and other high-glycemic CHO sources are easily digested, but fructose consumption should be minimized as it is absorbed at a slower rate and increases the likelihood of gastrointestinal problems.

• The addition of PRO (0.15 – 0.25 g PRO/kg/day) to CHO at all time points, especially post-exercise, is well tolerated and may promote greater restoration of muscle glycogen.

• Ingestion of 6 – 20 grams of EAAs and 30 – 40 grams of high-glycemic CHO within three hours after an exercise bout and immediately before exercise have been shown to significantly stimulate muscle PRO synthesis.

• Daily post-exercise ingestion of a CHO + PRO supplement promotes greater increases in strength and improvements in lean tissue and body fat % during regular resistance training.

• Milk PRO sources (e.g. whey and casein) exhibit different kinetic digestion patterns and may subsequently differ in their support of training adaptations.

• Addition of Cr to a CHO + PRO supplement in conjunction with regular resistance training facilitates greater improvements in strength and body composition as compared with when no Cr is consumed.

• Dietary focus should center on adequate availability and delivery of CHO and PRO. However, including small amounts of fat does not appear to be harmful, and may help to control glycemic responses during exercise.

• Irrespective of timing, regular ingestion of snacks or meals providing both CHO and PRO (3: 1 CHO: PRO ratio) helps to promote recovery and replenishment of muscle glycogen.

## Competing interests

The authors declare that they have no competing interests.

## Authors' contributions

CK – primarily responsible for drafting manuscript and incorporated revisions suggested by co-authors. TH, JS, BC, CW, RK, DK, TZ, HL, JL, JI, JA – All co-authors were equally responsible for writing, revising, and providing feedback for submission.  All authors reviewed content for scientific merit and provided general recommendations in relation to the direction of the manuscript.  All authors have read and approved the final manuscript.

## Supplementary Material

Additional file 1 Table 1 – Summary table of pre-exercise nutrition studies (Adapted from Hawley and Burke [22]). Click here for file

Additional file 2 Table 2 – Summary table of studies involving protein metabolism and nutrient timing after exercise. Click here for file

Additional file 3 Table 3 – Summary table of studies involving post-exercise nutrition administration and resistance training. Click here for file
