# Correction: International Society of Sports Nutrition position stand: Nutrient timing

**DOI:** 10.1186/1550-2783-5-18

**Published:** 2008-10-14

**Authors:** Chad Kerksick, Travis Harvey, Jeff Stout, Bill Campbell, Colin Wilborn, Richard Kreider, Doug Kalman, Tim Ziegenfuss, Hector Lopez, Jamie Landis, John L Ivy, Jose Antonio

**Affiliations:** 1 Department of Health and Exercise Science, University of Oklahoma, Norman, OK 73019, USA; 2 Endocrinology and Diabetes Section, Department of Pediatrics, University of Oklahoma Health Sciences Center, Oklahoma City, OK 73104, USA; 3 Center for Physical Development Excellence, Department of Physical Education, United States Military Academy, 727 Brewerton Road, West Point, NY 10996, USA; 4 School of Physical Education and Exercise Science, University of South Florida, Tampa, FL 33620, USA; 5 Exercise and Sport Science Department, University of Mary-Hardin Baylor, Belton, TX 76513, USA; 6 Department of Health and Kinesiology, Texas A&M University, College Station, TX 77843, USA; 7 Nutrition and Endocrinology Division, Miami Research Associates, Miami, FL 33143, USA; 8 Division of Sports Nutrition and Exercise Science, the Center for Applied Health Sciences, Fairlawn, OH 44333, USA; 9 Department of Physical Medicine and Rehabilitation, Northwestern University Feinberg School of Medicine, Chicago, IL 60611, USA; 10 Department of Biology, Lakeland Community College, Kirtland, OH 44094, USA; 11 Department of Kinesiology and Health Education, University of Texas, Austin, TX 78712, USA; 12 Farquhar College of Arts and Sciences, Nova Southeastern University, Fort Lauderdale, FL 33314, USA

## Correction

After publication of this work [1], we noted that we inadvertently failed to include the complete list of all coauthors. The full list of authors has now been added and the Authors' contributions and Competing interests section modified accordingly.

## Competing interests

The authors declare that they have no competing interests.

## Authors' contributions

CK was primarily responsible for drafting manuscript and incorporated revisions suggested by co-authors. All co-authors (TH, JS, BC, CW, RK, DK, TZ, HL, JL, JI, JA) were equally responsible for writing, revising, and providing feedback for submission. All authors reviewed content for scientific merit and provided general recommendations in relation to the direction of the manuscript. All authors have read and approved the final manuscript.
