# Supplementary material for: International Society of Sports Nutrition position stand: Nutrient timing
Source: J Int Soc Sports Nutr. 2008 Oct 3;5:17. doi: 10.1186/1550-2783-5-17 (PMC2575187; doi:10.1186/1550-2783-5-17)
Supplement: Additional file 1 — Table 1 – Summary table of pre-exercise nutrition studies (Adapted from Hawley and Burke [22]). [file 1550-2783-5-17-S1.doc]

Table 1: Summary table of pre-exercise nutrition studies (Adapted from Hawley and Burke [22])

| Authors [REF] | | Participants | Time before feeding (min) | | | CHO feeding | Exercise Regimen | Effect on Performance | | | |
| --- | --- | --- | --- | --- | --- | --- | --- | --- | --- | --- | --- |
| Chryssanthopoulos et al.[27] | | Five trained males  Four trained females | 30 | | | 75g glucose | Run to exhaustion at 70% VO_2_ max | No effect | | | |
| Devlin et al. [28] | | Eight untrained males | 30 | | | 43g sucrose | Cycle to exhaustion at 70% VO_2_ max | No effect | | | |
| Febbraio & Stewart [21] | | Six trained males | 45 | | | 1g CHO/kg BW as high or low glycemic index | Cycle for 2h at 70% VO_2_ max then 15min time trial | No effect | | | |
| Fielding et al. [96] | | Six trained males | 30 | | | 75 g glucose  75g fructose  150mL placebo | 30 min run at 70% VO_2_ max | No effect on glucose levels | | | |
| Foster et al. [23] | | Eight trained males  Eight trained females | 30 | | | 70g glucose | Cycle to exhaustion at 80% VO_2_ max | 19% decrease in time to exhaustion | | | |
| Gleeson et al. [97] | | Six untrained males | 45 | | | 70g glucose | Cycle to exhaustion at 70% VO_2_ max | 13% increase | | | |
| Goodpaster et al. [98] | | Ten trained males | 30 | | | 1g amylase or amylopectin starch/kg BW | Cycle 90 min at 66% VO_2_ max then 30 min time trial | 7% more work with amylopectin | | | |
| Hargreaves et al.[29] | | Six trained males | 45 | | | 75g glucose or 75g fructose | Cycle to exhaustion at 75% VO_2_ max | No effect | | | |
| McMurray et al. [30] | | Six trained females | 45 | | | 100g glucose or 100g fructose | Run to exhaustion at 80% VO_2_ max | No effect | | | |
| Okano et al. [24] | | Twelve trained males | 60 | | | 60-85g fructose | Cycle to exhaustion at 62-81% VO_2_ max | 7% increase | | | |
| Sherman et al. [25] | | Nine trained males | 60 | | | 75g glucose polymer or 150g glucose polymer | 90 min cycle at 70% VO_2_ max then time trial | 13% faster with CHO feedings | | | |
| Thomas et al. [26] | | Eight trained males | | | 60 | 70g CHO, as low GI or high GI | Cycle to exhaustion at 65-70% VO_2_ max | | | Low GI increased performance 20% |  |
| Smith et al. [99] | Ten trained males | | | 5 min  or 35 min | | 10% Glucose or Placebo | 4000m swim | | No sig. effect, however did ↑ times by 24s to 5 min. | | |
