# Supplementary material for: International Society of Sports Nutrition position stand: Nutrient timing
Source: J Int Soc Sports Nutr. 2008 Oct 3;5:17. doi: 10.1186/1550-2783-5-17 (PMC2575187; doi:10.1186/1550-2783-5-17)
Supplement: Additional file 2 — Table 2 – Summary table of studies involving protein metabolism and nutrient timing after exercise. [file 1550-2783-5-17-S2.doc]

Table 2: Summary table of studies involving protein metabolism and nutrient timing after exercise.

| Authors [REF] | Participants | Supplement/  Dosage | Time of Administration | Type of Exercise | Samples | Study Findings |
| --- | --- | --- | --- | --- | --- | --- |
| Biolo et al. [77] | 6 untrained males | - Infusion of balance AA mixture (0.15g•kg⁻¹•h⁻¹ for 3 h) | Two conditions:  - REST  - Post-ex. | - Leg resistance exercise routine | - Muscle biopsies  - Arteriovenous (A/V) blood sample for 3 h post-ex. | - PRO synthesis post-ex. > rest  - AA transport increased 30-100% post-ex. Compared to rest  - PRO intake immediately post-ex. May be more anabolic than other times |
| Borsheim et al. [78] | 16 (10M, 6F) recreationally active participants | Two conditions:  - 100g CHO  - Placebo (PLA) solution | Oral ingestion at 1h post-ex. | - Leg press (10 x 8 reps) @ 80% 1RM prior to 4 h bed rest  - 2 min. rest between sets | - A/V blood every 20 min. for 4 h post-ex.  - 4 biopsies (1 pre/3 post-ex.) | - CHO ingestion improved PRO balance after RE  - Effect, however, was minor and delayed compared with ingestion of AA |
| Tipton et al. [9] | 3M, 3F completed one PRE and POST session | EAC: 6g EAA + 35g CHO  PLA- Sweetened water | PRE: EAC solution pre-ex.  POST: EAC solution post-ex. | - Leg press and leg ext. 8-10 sets x 8 reps @ 80% IRM  - 2 min. rest between sets | - 16 venous and arterial blood samples pre-, during and post-ex over 180 min.  - Muscle biopsies @ 60, 0, 60 and 120 min. | - AA delivery increase w/ex. and 2h post-ex. in both trials  - PRO synthesis increased compared to post  - PRE AA uptake was increased 160% more than in POST  - EAC promoted positive N balance in both trials |
| Borsheim et al. [100] | 8 participants participated in both trials | Oral doses of:  - 77.4g CHO + 17.5g Whey + 4.9g AA (PAAC)  - 100g CHO (P) | 1 h post-exercise | - Resistance exercise | - Femoral artery,  vein and muscle  biopsies were  collected for 3 h  post-ex. | - PAAC caused an immediate increase and a delayed increase in protein balance  - PAAC stimulated a greater increase in PRO synthesis vs. CHO after resistance training. |
| Tipton et al. [79] | 3M, 3F | Three solutions:  - 40g CHO (PLA)  - 40g mixed AA  - 40g EAA | - Continual (100mL) consumption every 15-20 min. from 1 h post-ex. to 4 h post-ex. | - Leg Press (5 x 10 reps @ 75% IRM)  - Squat, leg curls/ext. (4 x 8 reps @ 75% 1RM | - Arteriovenous blood at 120, 30, 220, 260 and 270 min.  - Muscle biopsy was taken at 270 min. | - Both MAA and EAA increased net protein balance  - No difference in protein balance between MAA and EAA suggests no need for NEAA to cause protein synthesis |
| Tipton et al. [101]  *Table 2 (continued)* | 4F, 3 M | Two conditions  - REST  - REST + EAA + EX | - 15 g EAA solution before and after exercise session | - 8 x 8 reps @ 80% IRM  - 2 min. rest between sets | - Five biopsies and A/V blood samples were taken throughout entire 24 h protocol. | - AA exchanges (ES-REST) @ 3 h and 24 h was not different  - Acute response of muscle to EAA intake + EA is additive to rest and thus reflects 24 h response. |
| Tipton et al. [31] | 23 healthy untrained young males and females | Three conditions:  - Placebo (PLA)  - Casein (CAS)  - Whey protein  (WP) | 1 h post-ex. | - Leg ext. 10 x 8 reps @ 80% IRM  - 2 min. rest  between sets | - Femoral A/V blood samples for 3 h post-ex.  - 4 biopsies (pre-, 1h, 2h, 5h post-ex.) | - Both WP and CAS increased PRO balance to promote PRO synthesis  - AA uptake was increased after exercise in both groups |
| Rasmussen et al. [83] | 3M, 3F in postabsorptive state and recreationally active | - Placebo drink (PLA)  - EAA+CHO drink (35g CHO+6g EAA) | Two conditions:  -PLA 1h post-ex + EAA+CHO 3h post-ex  - EAA+CHO 1h post-ex. + PLA 3h post-ex. | - 8-10 sets x 8 reps @ 80% 1RM  - 2 min. rest between sets | - 11 A/V blood over 7 h time span.  - Muscle biopsies at 45 min. 2 h and 4h post-ex | - No change in PLA  - EAA+CHO increased PRO balance and PRO SYN 1 h and 3 h post-ex.  - No increase in PS and PRO balance at 3 h vs. 1 h with both >than pre-ex. |
| Borsheim et al. [74] | 3M, 3F recreationally active | 6g EAA | - 2 oral doses of 6g EAA at 1 h and 2 h post-ex | - 8-10 sets x 10 reps of leg press and leg ext. @ 80% 1RM  - 2 min. rest between sets | - 4 muscle biopsies were provided post-ex  - Femoral artery and venous blood samples every ~20 min. for 4 h post-ex. | - PRO balance increased in response to both drinks and decreased when [AA] returned to basal levels  - Non-essential AA are not necessary for increasing PRO balance  - PRO synthesis is dose-dependent of [EAA] |
| Boirie et al. [92] | 16 young (24±4 years) healthy participants | - 30 g whey  - 43 g casein (equal [Leu])  - 30 g unlabeled whey  - 30 g unlabeled casein  - 43 g unlabeled casein | - Feeding trial  - No exercise  - All conditions were ingested at same time | NONE | - A/V blood samples for entire 7 h time period | - CAS had prolonged plateau of high [AA] inhibiting PRO breakdown by 34%  - WP caused dramatic increase in [AA] causing 68% increase in PRO synthesis  - Speed of PRO digestion and AA absorption have major effect on protein synthesis. |
| Dangin et al. [93] | 22 healthy young males (25±1 yrs) | - 30g casein  - 30g free AA mimic casein  - 30g whey  - Repeated small doses of whey | - Feeding trial  - No exercise | NONE | - A/V blood samples for entire 7 h time period | - “Fast” meals (whey and free AA) caused strong, rapid increase of [AA]  - Moderate increase in [AA] with “slow” meals and stayed elevated longer  - 7 h Leu balance was higher after slow vs. fast meals |

*Table 2 (continued)*
