# Supplementary material for: International Society of Sports Nutrition position stand: Nutrient timing
Source: J Int Soc Sports Nutr. 2008 Oct 3;5:17. doi: 10.1186/1550-2783-5-17 (PMC2575187; doi:10.1186/1550-2783-5-17)
Supplement: Additional file 3 — Table 3 – Summary table of studies involving post-exercise nutrition administration and resistance training. [file 1550-2783-5-17-S3.doc]

Table 3: Summary table of studies involving post-exercise nutrition administration and resistance training.

| Authors [REF] | Participants | Supplement/  Dosage | Time of Administration | Type of Exercise | Samples | Study Findings |
| --- | --- | --- | --- | --- | --- | --- |
| Hartman  et al.[86] | 56 M | Fat-free milk  Fat-free soy  Maltodextrin (CON) | 1^st^ dose: Immediately after  2^nd^ dose: 1 h post-ex | RT 5 days/wk for 12 weeks | Strength  Body Comp (DXA), Fiber size | - Increased strength and body comp  - Type II area > in Milk vs. Soy & CON  - Type I area > in Milk vs. CON  - DXA FFM > in Milk vs. Soy & CON |
| Cribb & Hayes [8] | 23 recreationally trained males | - 40g PRO/43g CHO/6g Cr per 100 g  - Delivered at dose of 1g/kg BM  - Training days only | Two groups:  1) Dose 1 in morning; Dose 2 in evening  2) Dose 1 pre ex; Dose 2 post ex | Whole-body RT program | 1RM strength  Body comp (DXA)  Muscle CSA | - Pre-Post reported greater increases in lean mass and 1RM along with greater CSA changes in type II fibers |
| Kerksick  et al. [87] | 36 resistance trained males | - 48g CHO  - 40g whey PRO + 8g casein PRO  - 40g whey PRO + 5g glutamine + 3g BCAA | Post-exercise | 10 weeks; split-body RT program 4 days/wk using 6-10 RM loads | 1RM strength  Body comp (DXA)  Anaerobic power | - Increases in 1RM for all groups  - DXA lean and fat-free mass greatest in PRO + CAS |
| Esmarck  et al. [102] | 13 M older individuals (74±1 yrs) | - Liquid supplement (10g PRO + 7g CHO + 3g fat) | - Immediately post training session (P0)  - 2 hr post training session (P2) | - 12 week RT program  (3x/week) | - Body comp. (DEXA)  -Hypertrophy via MRI and muscle biopsies  - Isokinetic strength | - P0 > P2 for cross-sectional area and mean fiber area  - Similar change seen in strength  - Immediate protein supplementation post-exercise promotes more growth in elderly males |
| Willoughby  et al. [10] | 19 untrained males | 20g PRO or 20g CHO  2 doses/day | 1 h before and 1 h after exercise | 10 weeks of RT; 4x/wk @ 85-90% 1RM | - 1RM strength  - Body comp  - MHC and IGF-1 expression | - Greater increases in body mass, fat-free mass, thigh mass, strength, serum IGF-1, IGF-1 mRNA, MHC I and IIa expression and myofibrillar protein |
| Cribb  et al. [84] | 33 male recreational body builders | Cr + CHO  Cr + Whey PRO  Whey PRO  CHO | 3 equal servings:  - Midmorning  - Post-workout  - Evening | 11 weeks of RT | 1RM strength  Body comp (DXA)  Fiber CSA  Protein content | Cr + CHO, WP and Cr + WP increased strength and hypertrophy vs. CHO |
| Cribb et al. [85] | 31 male recreational body builders | PRO  PRO + CHO  PRO + CHO + Cr | 3 equal servings:  - Midmorning  - Post-workout  - Evening | 10 weeks of RT | 1RM strength  Body comp (DXA)  Fiber CSA  - Protein content | PRO + CHO + Cr increased 1RM and lean body mass, fiber CSA and protein content vs. PRO + CHO |
| Kerksick et al. [88] | 49 M & F trained participants | PRO  PRO + Colostrum  PRO + Cr  Colostrum + Cr | Post-exercise ingestion | 12 weeks; split-body RT 4 days/wk using 6-10 RM loads | 1RM strength  Body comp (DXA)  Anaerobic power | - In comparison to PRO, PRO + Col, PRO + Cr, Col + Cr increased body mass and DXA scanned mass  - PRO + Cr and Col + Cr had greater increased FFM vs. PRO + Col |
| Tarnopolsky et al. [90] | 19 healthy males | CHO + Cr  PRO + CHO + Cr | Post-exercise ingestion | 8 weeks of RT; 1h/d for 6 d/wk | 1RM strength  Body comp (DXA)  Fiber area | Similar changes in strength and body comp between groups; greater increase in body mass for PRO + CHO + Cr |
